# Peripancreatic pseudoaneurysms: a management-based classification system

**DOI:** 10.1007/s00464-014-3434-9

**Published:** 2014-02-12

**Authors:** Tony C. Y. Pang, Richard Maher, Sivakumar Gananadha, Thomas J. Hugh, Jaswinder S. Samra

**Affiliations:** 1 Upper GI Surgical Unit, Royal North Shore Hospital and North Shore Private Hospitals, University of Sydney, St Leonards, NSW 2065 Australia; 2 Department of Radiology, Royal North Shore Hospital and North Shore Private Hospitals, University of Sydney, St Leonards, NSW 2065 Australia; 3 Discipline of Surgery, Sydney Medical School, University of Sydney, Sydney, 2000 Australia

**Keywords:** Pseudoaneurysm, Pancreatoduodenectomy, Chronic pancreatitis, Acute pancreatitis

## Abstract

**Background:**

Peripancreatic pseudoaneurysms can arise in a number of different clinical settings but are associated mostly with pancreatitis and pancreatobiliary surgery. The aim of this study is to review the current literature and to propose a management classification system based on the pathophysiological processes and the exact anatomical site of peripancreatic pseudoaneurysms.

**Methods:**

A systematic review of the literature from 1995 to 2012 was performed. Articles on studies describing peripancreatic pseudoaneurysms in the setting of pancreatitis or major hepatic or pancreatic surgery with more than ten patients were included. Seventeen eligible studies were identified and reviewed.

**Results:**

The demographic characteristics of the patients in all studies were similar with a predominance of males and a mean age of 55 years. The overall mortality rate varied greatly among the studies, ranging from 0 to 60 %. Embolisation was the first line of management in the majority of the studies, with surgery reserved for failed embolisation or for haemodynamically unstable cases. Embolisation of the hepatic artery or its branches was associated with high rates of morbidity (56 %) and hepatic failure (19 %). More recent studies show that stents are used increasingly for vessels that cannot be embolised safely. Late bleeding, a major cause of mortality and morbidity, is generally underreported. The proposed classification system is based on three factors: (1) the type of artery from which the pseudoaneurysm arises, (2) whether communication with the gastrointestinal tract is present, and (3) whether there is high concentration of pancreatic juice at the bleeding site.

**Conclusion:**

The management of peripancreatic pseudoaneurysms usually comprises a combination of interventional radiology and surgery and this may be assisted by a logical classification system.

Managing peripancreatic pseudoaneurysms is complex and challenging. Advances in imaging technology and interventional radiology have had an enormous impact on both the diagnosis and the treatment of this condition. Pseudoaneurysms can arise in a number of different clinical settings but are associated mostly with pancreatitis and pancreatobiliary surgery [1].

Initially, the high mortality associated with this condition is due to uncontrolled torrential bleeding. If the patient survives this initial phase there is a risk of rebleeding, which can occur from days to years after the initial presentation. The additional late mortality may be substantial if patients are not managed appropriately during their initial presentation. Treatment strategies should be based on the mechanisms that lead to the formation of pseudoaneurysms and to the ongoing pathophysiological processes that result in rebleeding.

Currently, the best method of assessing a relatively stable patient is a computerised tomography (CT) angiogram with or without a formal angiogram [2]. This usually provides an accurate diagnosis but also allows, if required, radiological intervention by embolisation of the feeding vessel or the pseudoaneurysm itself. If visceral ischaemia distal to the pseudoaneurysm is a concern, a stent may be placed [1, 3]. Patients with haemodynamic instability may need immediate surgical intervention, although this can be fraught with danger even in experienced hands.

There are no data on definitive long-term management strategies for this condition. This is partly due to its low rate of occurrence but also because of a poor understanding of the pathophysiological processes involved. The mechanisms by which most peripancreatic pseudoaneurysms are formed are thought to be related to the underlying inflammation, the presence of pancreatic juices, and the subsequent infection [4]. In postoperative patients, pseudoaneurysms are often associated with intra-abdominal sepsis due to anastomotic leakage [5]. Unless these underlying processes are dealt with adequately, the risk of further bleeding can be substantial.

The aim of this article is to review the literature regarding pancreatic pseudoaneurysms with an emphasis on current treatment practices. We also propose a management classification system based on the pathophysiological processes and the exact anatomical site of the pseudoaneurysm. A classification such as this may help with decision making during initial and definitive management to minimise the risk of rebleeding and to reduce late mortality.

## Methods

### Search strategy

A literature search was performed using “keywords” and “headings” in Medline and EMBASE limited to publications from 1995 to 2011. Keyword searches of panc*, surg*, resect*, and neoplasm* and heading searches of pseudoaneurysm, pancreatitis, operation, cancer, and neoplasm were used along with Boolean operators.

Due to the variability of the descriptions of peripancreatic arterial haemorrhagic complications, articles were eligible only if they included patients with peripancreatic pseudoaneurysms, “aneurysms,” or peripancreatic arterial bleeding detected on angiography. Non-English articles, single case reports, case series with fewer than ten patients (even if more than ten pseudoaneurysms were treated), and reviews were excluded. Case series with patients with mixed pathologies (e.g., haemosuccus pancreaticus, suspected pancreatic bleeding with no definitive angiographic findings [6], or delayed postpancreatectomy haemorrhage) were included only if adequate clinical information about patients with the above eligible pathology could be extracted or if patients were selected such that only those with visceral arterial bleeding was included. Articles describing visceral pseudoaneurysms that were not associated with pancreatitis or pancreatobiliary surgery (e.g., vasculitis) were also excluded [7].

Twenty studies were identified during the initial literature search and 17 met the eligibility criteria. Two pairs of studies were most likely from the same institution and therefore only the more recent study was included in the analysis [8, 9]. One additional study was excluded due to insufficient information [10].

### Statistical analysis

All statistical analyses and charting were performed with Microsoft Excel 2010 for Windows (Microsoft Corp., Redmond, WA) and Stata SE version 11.2 for Windows (StataCorp., College Station, TX). Due to the heterogeneity of the patient population and the treatment strategies, no inferential statistical analyses were performed. Only descriptive statistical analyses are presented and discussed.

## Results and discussion

Pancreatic pseudoaneurysms are relatively rare and the exact incidence is difficult to measure due to the varying aetiologies. The most common associations are with pancreatitis and major pancreatobiliary surgery. The relative incidence of these two aetiologies is not clear because a significant number of studies present either type but not both. Pseudoaneurysms can occur in both acute and chronic pancreatitis; however, they are more common in chronic pancreatitis and are often associated with pseudocysts [11]. It is thought that pancreatic juice within the pseudocyst causes enzymatic degradation of the adjacent arterial wall, with weakness and rupture leading to pseudoaneurysm formation. In the setting of major pancreatobiliary surgery, pseudoaneurysm formation is thought to be associated with either skeletonisation of vessels or complicating bilio- or pancreaticoenteric anastomotic leak [5, 12, 13].

The studies reviewed are listed in Table 1. The demographic characteristics of the patients in all studies were similar, with a predominance of males and a mean age 55 (45–66) years (excluding the studies De Perrot et al. [14] and Lee [15]). The mean age of the surgical patients who developed pseudoaneurysms was older than that of the patients with pancreatitis (range of means: 58–66 vs. 44–57 years). Interestingly, young patients (<40 years) were almost exclusively in the pancreatitis group. This age difference was confirmed by Zyromski et al. [3]; theirs was the only study that compared these two patient groups (mean age = 62 vs. 46, *P* < 0.007).

**Table 1:** Demographic and mortality data

	Postop^a^	Pancr^a^	Total^a^	Year	Age^b^	Sex (M:F)	Presentation^c^				Sentinel bleed^c^		Mortality^c,g^
							Intra-abdominal bleed^d^	GI bleed^e^	Occult/shock^f^	Pain			
*Postoperative only*													
Okuno et al. [13]	14	0	14	1985–1998	60 (40–72)	11:3	10 (71)	4 (29)	–	10 (71)	11 (79)		4 (29)
Fujii et al. [12]	13	0	13	1993–2005	66 (SD 10)	10:3	–	–	–	–	–		7 (54)
Miura et al. [5]	15	0	15	1981–2007	65 (50–82)	11:4	–	–	–	–	7 (47)		9 (60)
Lee [15]	27	0	27	1995–2007	–	–	13 (48)	10 (37)	6 (22)	–	21 (78)		6 (22)
Schäfer et al. [30]	18	0	18	1998–2009	58 (39–82)	11:7	4 (22)	7 (39)	9 (50)	2 (11)	–		6 (33)
Lee et al. [31]	27	0	27	2003–2011	64 (48–86)	20:7	20 (74)	13 (48)	–	–	22 (82)		6 (22)
*Pancreatitis only*													
Gambiez et al. [37]	0	14	14	1983–1994	46 (32–60)	11:3	2 (14)	8 (57)	4 (29)	–	N/A		2 (14)
De Perrot et al. [14]	0	10	10	1978–1997	44 (med) (28–61)	7:3	–	–	9 (90)	10 (100)	N/A		1 (10)
Carr et al. [38]	0	16	16	1988–1998	45 (23–67)	11:5	–	–	–	–	N/A		3 (19)
Beattie et al. [23]	0	13 ^h^	13	1995–1999	57 (25–81)	11:8	–	–	–	–	N/A		3 (21)
Bergert et al. [11]	0	35	35	1993–2004	51 (28–75)	28:7	–	16 (46)	15 (43)	35 (100)	N/A		7 (20)
Zhou et al. [40]	0	19	19	2000–2003	43 (26–61)	10:9	–	–	5 (26)	–	–		–
Lermite et al. [16]	0	17	17	1981–2005	57 (35–70)	15:2	–	13 (76)	2 (12)	2 (12)	N/A		0 (0)
Udd et al. [25]	0	33	33	1993–2005	54 (42–78)	27:6	–	17 (52)	–	22 (67)	N/A		2 (6)
Sethi et al. [27]	0	16	16	2000–2007	52 (21–71)	11:5	2 (13)	8 (50)	10 (63)	14 (88)	N/A		0 (0)
*Both pancreatitis and postoperative*													
Zyromski et al. [3]	13	24	37	1995–2005	Postop 62 (SD 15) Pancr 46 (SD 11)	21:16	7 (19)	14 (38)	3 (8)	15 (41)	12/13 (92)		Postop 4 (31) Pancr 1 (4)
Kalva et al. [1]^j^	9	12	23^i^	1978–2010	64 (21–84)	17:6	19 (83)	2 (9)	2 (9)	–	6 (26)		

*GI* gastrointestinal, *N/A* not applicable, *Pancr* pancreatitis, *Postop* postoperative, *SD* standard deviation, – no data, *med* median

^a^Values are number (*n*)

^b^Values are mean (range)

^c^Values are number (percent)

^d^Intra-abdominal bleed includes bleeding from drain as presenting symptom and massive distention

^e^Gastrointestinal bleed includes haematemesis, melaena, and haematochezia

^f^Occult/shock is no obvious presenting symptoms apart from signs of blood loss, either acute or chronic (shock, anaemia)

^g^Mortality is 30-day or inpatient all-cause mortality

^h^Article described 19 patients, but only 13 demonstrated angiographic evidence of bleeding or pseudoaneurysm (1 vasculitis, 5 negative angiograms). Age and sex distribution based upon the entire 19 patients described in the paper

^i^Two patients were secondary to trauma

^j^Kalva et al’s study described 51 patients, but only 23 patients demonstrated a pseudoaneurysm or evidence of active extravasation

### Presentation and outcome

Pseudoaneurysms may rupture into the gastrointestinal tract (GIT), the peritoneal cavity, the pancreatic duct, or a pseudocyst. Bleeding into the GIT can occur directly or indirectly. The latter is often due to haemosuccus pancreaticus or haemobilia [3, 16], where patients present with haematemesis, haematochezia, or melaena. Those with extensive retroperitoneal haematoma, but without bleeding into the GIT, may present with pain only. In the studies reviewed, gastrointestinal bleeding was the most common presentation overall. In postoperative patients, intra-abdominal bleeding was the most common presentation, reflecting the underlying pathophysiology. On the other hand, in patients with pancreatitis, pain was the most prominent presenting symptom (Table 1).

The onset of fresh bleeding from a drain following pancreatic surgery is an ominous sign suggestive of intra-abdominal bleeding, including from a pseudoaneurysm. It is known as a sentinel bleed. Initial minor bleeding may be followed by more catastrophic blood loss and this occurs in up to 90 % of cases [5, 15]. The importance of a sentinel bleed was first highlighted by Sato et al. [17] who described it in all ten patients in their study with massive bleeding following pancreatectomy. More recent studies also suggested that this is frequently a warning sign of imminent and massive haemorrhage after pancreaticobiliary surgery [18, 19]. Early angiography is recommended as this may allow intervention while the patient is still haemodynamically stable. On the other hand, some authors have found that despite awareness of this entity, identification of a sentinel bleed was not associated with a significant difference in mortality [20]. Unfortunately, it is unclear from this latter study whether specific interventions were undertaken in response to the bleeding. Overall, sentinel bleeding was noted in 47–92 % of patients in the studies reviewed (Table 1).

The overall mortality rate in patients with bleeding peripancreatic pseudoaneurysms varied greatly among the studies, ranging from 0 to 60 %. The mortality rate for bleeding associated with pancreatitis (0–21 %) was lower compared with that for bleeding post-surgery (22–60 %). Unfortunately, few studies included patients from both groups, which prevented meaningful inferential statistical analysis. The study by Zyromski et al. [3] was the only one that compared the two groups and, indeed, found a statistically significant difference in mortality favouring the pancreatitis group (31 % vs. 4 %, *P* = 0.04). In the postpancreatitis group, there was a trend toward improvements in mortality, with the most recent studies reporting mortality rates of less than 10 %. This compares favourably with historical mortality rates (up to 30 %) for patients who underwent operative intervention for postpancreatitis pseudoaneurysms [21, 22].

### Treatment strategies

Initial control of the pseudoaneurysm or any active bleeding may be achieved radiologically or surgically. The range of surgical and nonsurgical treatment options and outcomes is summarised in Tables 2 and 3. In studies that did not select patients based on treatment strategy, embolisation was the first line of management in the majority of them published after 2000 (46–92 %) [3, 11]. While some authors preferred angiography (with or without embolisation) as the initial management option [1, 23], others were more selective depending on the presentation [24]. This may be a reflection of local expertise and resource availability.

**Table 2:** Nonsurgical treatments and outcomes

		Selective?^a^	Angiographic management *n* (%)										No intervention *n* (%)		
			TAE	Stent		Failures	Rebleed	Secondary treatment				Death		Indication	Death
	A		B	C		D	E	F				G	*N*		
	*n*		*n* (% A)	*n* (% A)	Vessel stented	*n* (% B + C)	*n* (% B + C)	Emb	Sten	Sur	No	*n* (% B + C)	*n* (% A)		*n* (% *N*)
*Postoperative only*															
Okuno et al. [13]	14	–	12 (86)	0 (0)		2 (17)^b^				1	1	3 (25)	1 (7)^c^	SMA	0 (0)
Fujii et al. [12]	13	–	9 (69)	0 (0)		4 (44)^b^		2		2		4 (44)	1 (8)	Occlusion CHA^d^	1 (100)
Miura et al. [5]	15	–	12 (80)	0 (0)		1 (8)	1 (8)	1			1	6 (50)	0 (0)		
Lee [15]	27	–	22 (81)	1 (4)	SMA	5 (22)	4 (17)	4		5		3 (13)	2 (7)	Death	2 (100)
Schäfer et al. [30]	18	–	9 (50)	3 (17)^f^	–	2 (17)	3 (25)^g^	3		1	1	2 (17)	0 (0)		
Lee et al. [31]	27	–	22 (81)	4 (15)	–	1 (4)	0 (0)			3^e^		5 (19)	0 (0)		
*Pancreatitis only*															
Gambiez et al. [37]	14	–	14 (100)	0 (0)		3 (21)	0 (0)			3		2 (14)	0 (0)		
De Perrot et al . [14]	10	–	3 (30)	0 (0)		0 (0)	1 (33)				1	1 (33)	0 (0)		
Carr et al. [38]	16	–	6 (38)	0 (0)		3 (50)	0 (0)			3		0 (0)	0 (0)		
Beattie et al. [23]	13	–	8 (62)	0 (0)		0 (0)	3 (38)	1		1	1	1(13)	2 (15)	Unknown	0 (0)
Bergert et al. [11]	35	–	16 (46)	0 (0)		0 (0)	2 (13)	2				3 (19)	0 (0)		
Zhou et al. [40]	19	T	19 (100)	0 (0)		2 (11)	7 (37)			2		5 (26)	0 (0)		
Lermite et al. [16]	17	–	8 (53)	0 (0)		2 (22)	3 (33)	3		2		0 (0)	1 (6)	Angio –ve	0 (0)
Udd et al. [25]	33	A	23 (70)	0 (0)		4 (17)^b^		4				1 (4)	0 (0)		
Sethi et al. [27]	16	–	13 (82)	0 (0)		3 (23)	0 (0)^h^	3				0 (0)	1 (6)	Spont thromb^i^	0 (0)
*Both pancreatitis and postoperative*															
Zyromski et al. [3]	37	–	34 (92)	1 (3)	–	0 (0)	2 (6)	2				4 (11)	1 (3)	Unknown	–
Kalva et al. [1]	23	A	23 (100)	0 (0)		1 (4)	4 (17)	2	1		2	6 (26)	0 (0)		

*A/Angio* angiogram, *CHA* common hepatic artery, *Emb* embolisation, *No* no interventional treatment, *Sten* vascular stent insertion, *Surg* surgical management, *SMA* superior mesenteric artery, *Spont thromb* spontaneous thrombosis, *T* trans-arterial embolisation

^a^Selective group by intervention: *T* TAE, *A* angiogram, – not selected on basis of intervention

^b^Unclear whether it is primary failure or recurrent bleeding after initial haemostasis

^c^Intra-arterial vasopressin infusion for SMA bleed (TAE not performed for fear of ischaemia)

^d^Intimal injury to CHA during angiography → occlusion and control of PHA extravasation

^e^Three patients required secondary procedure for abdominal compartment syndrome. The one patient with failed embolisation sustained a CHA dissection, which actually controlled the bleeding due to reduced intrahepatic arterial flow

^f^These three patients also had coiling performed

^g^The number of failures/rebleeding and the subsequent intervention is unclear from the paper

^h^Three patients with continued filling of the pseudoaneurysm on CT. These were embolised successfully

^i^Spontaneous thrombosis of a gastroduodenal pseudoaneurysm

**Table 3:** Surgical treatments and outcomes

	Surgical management												
	A	Surgery	Indication				Failure	Rebleed	Secondary treatment				Death
		I					J	K					M
	*n*	*n* (% A)	Emer	Tech^a^	Isch	Other	*n* (% I)	*n* (% I)	Emb	Sten	Sur	No	*n* (% I)
*Postoperative only*													
Okuno et al. [13]	14	1 (7)		1			0 (0)	0 (0)					1 (100)
Fujii et al. [12]	13	3 (23)	1		2		0 (0)	0 (0)					2 (66)
Miura et al. [5]	15	3 (20)	2		1		1 (33)	0 (0)				1	3 (100)
Lee [15]	27	2 (7)	2				0 (0)	1 (50)				1	1 (50)
Schäfer et al. [30]	18	6 (39)	2			4	1 (17)	3 (50)^b^					4 (67)
Lee et al. [31]	27	1 (4)	1				0 (0)	0 (0)					1 (100)
*Pancreatitis only*													
Gambiez et al. [37]	14	0 (0)					–	–					–
De Perrot et al. [14]	10	7 (70)	3			4^c^	0 (0)	0 (0)					0 (0)
Carr et al. [38]	16	10 (63)	6			4	3 (30)	1 (10)	4				3 (30)
Beattie et al. [23]	13	4 (31)	3	1			0 (0)	0 (0)					0 (0)
Bergert et al. [11]	35	19 (54)	9			10	3 (16)	4 (21)	No data				4 (21)
Zhou et al. [40]	19	0 (0)					–	–					–
Lermite et al. [16]	17	7 (41)	7				0 (0)	0 (0)					0 (0)
Udd et al. [25]	33	10 (30)		10			0 (0)	1 (10)	1				1 (10)
Sethi et al. [27]	16	2 (13)	2				2 (100)	0 (0)	1		1		0 (0)
*Both pancreatitis and postoperative*													
Zyromski et al. [3]	37	1 (3)	1				1 (100)	0 (0)				1	1 (100)
Kalva et al. [1]	23	0 (0)					–	–					–

*Emb* embolisation, *Emerg* emergency surgery, *Isch* fear of ischaemia, *Stent* vascular stent, *Sur* surgical management, *No* no management

^a^Technical failure includes inability to access or visualise vessel on angiogram and other technical reasons for failure of angioembolisation

^b^Number of failures/rebleeds unclear from paper. This is the best estimate from text

^c^Operative management was standard treatment at the time

In postoperative studies, mortality rates in the surgical group were generally higher (50–100 %) than those in the embolisation group (13–50 %). However, this might be expected given that patients requiring early surgical intervention are usually more haemodynamically unstable than those who can wait for angiography.

In the pancreatitis studies, outcomes for surgical and nonsurgical intervention were similar except in the earlier studies. Specifically, the study by De Perrot et al. [14], published in 1999, stood out in that it demonstrated a high mortality rate in patients who were embolised (33 %) compared with a zero mortality rate for the surgical patients. This may reflect relative inexperience with embolisation techniques at a time when surgical therapy was the standard approach.

Obviously, patient selection and timing play important roles in determining the morbidity and mortality of either procedure. Bergert et al. [11] demonstrated this by dividing their patients into those requiring urgent and those requiring semiurgent intervention. Three of nine (33 %) urgent surgical patients died compared with only one of ten (10 %) semiurgent patients. Herein lies the difficulty in comparing outcomes of surgical and embolisation groups in these case series where the indications and underlying pathologies for embolisation and surgery may be different. This makes statistical comparisons between the outcomes of these two modalities difficult and probably unnecessary. Nonetheless, some authors have attempted statistical analyses. For example, Udd et al. [25], in a series of 33 pseudoaneurysmal bleeds from chronic pancreatitis, found no difference in morbidity and mortality between the embolisation and surgical groups. Similarly, Roulin et al. [26] performed a meta-analysis comparing laparotomy and interventional radiology for delayed postoperative haemorrhage following pancreatic surgery and, not surprisingly, found a higher mortality rate in the surgical group.

The overall reported success rates of embolisation are high, but this often includes multiple embolisations as well as prolonged supportive treatment. As a single procedure, embolisation alone has a substantial failure rate. The primary failure rate in the studies reviewed ranged from 0 to 50 %, with a rebleeding rate of 0–38 % (Table 2). Overall, this translates into a total failure rate of between 6 and 55 %, with half of the studies (median of all studies) having a failure rate of greater than 23 %. Of the combined 262 patients embolised or stented in all reviewed studies, 51 required a secondary procedure (either re-embolisation or surgery).

Although embolisation was used as first line treatment in most of the recent studies, surgery still remains an important treatment modality, especially in the setting of a haemodynamically compromised patient or when angiographic management fails. The low operation rates in some studies suggest that haemodynamically compromised patients may still be treated radiologically at first [3, 13, 15, 27]. For this to work optimally, the initial radiological intervention should be carried out in an angio-theatre suite, so if it fails, immediate surgical intervention is possible. Other indications for surgery included situations where access to the bleeding vessel was impossible radiologically and when there was failed visualisation or failed embolisation of the bleeding vessel for definitive management of the underlying cause or for other coexisting abdominal pathologies [3, 25, 27]. The indications and outcomes of operative management in the studies reviewed are summarised in Table 3.

The use of stents is a third treatment modality that is being used increasingly for visceral pseudoaneurysms [28, 29]. A stent has the advantage of excluding the pseudoaneurysm while allowing continued flow through the feeding vessel. Only four of the case series reviewed used stents to achieve haemostasis. Lee [15] placed stents in both the common hepatic artery (CHA) (for a short gastroduodenal stump pseudoaneurysm) and the superior mesenteric artery (SMA) in an attempt to avoid end organ ischaemia. Zyromski et al. [3] placed a stent in one patient, but it is unclear in which vessel or the specific indication. The two most recent studies reviewed [30, 31] employed arterial stents as primary intervention for 15 and 17 % of patients, respectively. However, no details were given as to which vessel was stented or the indications. Satisfactory results with stents have been reported by small selective case series. Herzog et al. [29] reported four patients in whom successful haemostasis was achieved with covered stents used for delayed visceral haemorrhage following pancreatic surgery. Similarly, successful haemostasis was achieved in all patients in two smaller case series of four and five patients with hepatic arterial bleeding [28, 32].

Visceral artery stent placement, however, is not without potential complications. The long-term patency and clinical outcomes of stents in this setting are not known [32]. Some authors argue that stent occlusion from intimal hyperplasia may not affect long-term outcome given that it is a slow process which allows time for the formation of collaterals [33]. Such collateralisation did not develop in a patient reported by Lee [15]. The patient developed major thrombosis of the SMA after stent placement resulting in long-term parenteral nutrition due to short-gut syndrome. Other disadvantages of this technique include the possibility of stent infection, exclusion of branches close to the pseudoaneurysm, kinking or misplacement of the stent, and arterial rupture during placement. The lack of availability of small visceral artery “covered” stents may also be a limitation to this approach [5]. The potential complication of stent infection [34] is a major concern in this setting where frequently there is communication with the GIT or the presence of infected necrotic tissue (e.g., necrotising pancreatitis). Infection of a foreign body, such as a stent, may become chronic due to ongoing contamination from an uncontrolled gastrointestinal anastomotic leak. This may be exacerbated if there is a pancreatic anastomotic breakdown where the digestive enzymes contribute to the degradative process.

### Influence of vessel type on treatment strategy

The distribution of involved arteries in each study is summarised in Table 4. As expected, pancreatitis-related pseudoaneurysm formation and bleeding involve most commonly either the splenic artery or the gastroduodenal/pancreaticoduodenal arterial complex. Post pancreatic surgery cases can affect the same vessels, but the hepatic and mesenteric arteries may also be at risk as a result of an anastomotic leak or an operative injury during radical lymphadenectomy. Unfortunately, it is impossible to glean from the reviewed studies the exact cause of the bleeding and its relationship to arterial distribution.

**Table 4:** Distribution of artery origins of the pseudoaneurysms in each study

	Bleeding point/feeding vessel (main vessel or branches of)						Total with PA or bleeding point^a^
	SPA	GDA/PDA	CHA/PHA	L/R/M HA	SMA	Other	
*Postoperative only*							
Okuno et al. [13]	0 (0)	3 (21)	0 (0)	8 (57)	1 (7)	2 (14)	14
Fujii et al. [12]*	2 (14)	5 (36)	2 (14)	4 (29)	1 (7)	0 (0)	14
Miura et al. [5]	0 (0)	6 (38)	4 (25)	2 (13)	4 (25)	0 (0)	16
Lee [15]	1 (4)	12 (48)	4(16)	5 (20)	1 (4)	2 (8)	25
Schäfer et al. [30]	4 (22)	2 (11)	7 (39)	3 (17)	1 (6)	1 (6)	18
Lee et al. [31]	No data						27
*Pancreatitis only*							
Gambiez et al. [37]	4 (29)	8 (57)	0(0)	0 (0)	0 (0)	1 (7)	14
De Perrot et al. [14]	6 (60)	3 (30)	0 (0)	0 (0)	0 (0)	1 (10)	10
Carr et al. [38]	3 (23)	9 (70)	0 (0)	0 (0)	1 (8)	0 (0)	13^b^
Beattie et al. [23]	3 (23)	8 (62)	0 (0)	0 (0)	0 (0)	2 (15)	13
Bergert et al. [11]	10 (27)	16 (43)	4 (11)	0 (0)	4 (11)	3 (8)	37
Zhou et al. [40]				No data			19
Lermite et al. [16]	6 (35)	8 (47)	1 (6)	0 (0)	1 (6)	1 (6)	17
Udd et al. [25]	14 (42)	19 (58)	0 (0)	0 (0)	0 (0)	0 (0)	33
Sethi et al. [27]	7 (44)	3 (19)	3 (19)	0 (0)	0 (0)	3 (19)	16
*Both pancreatitis and postoperative*							
Zyromski et al. [3]	14 (36)	15 (38)	5 (13)	0 (0)	3 (8)	2 (5)	39
Kalva et al. [1]	5 (21)	10 (42)	3 (13)	2 (8)	0 (0)	4 (17)	24

*CHA* common hepatic artery, *GDA* gastroduodenal artery, *L/R/MHA* left/right/middle hepatic artery, *PA* pseudoaneurysm, *PDA* pancreatoduodenal artery, *PHA* proper hepatic artery, *SMA* superior mesenteric artery, *SPA* splenic artery

^a^Some patients had multiple PA so the total number of PA may be different from the total number of patients in the study

^b^Three patients’ bleeding point unknown

* Three patients did not demonstrate pseudoaneurysm on angiogram but rather had extravasation in the area of the respective vessels

Splenic, gastroduodenal, and pancreaticoduodenal arteries were the most commonly involved vessels in the studies reviewed. Embolisation of the splenic artery was relatively safe, with only four patients reported to have suffered splenic infarction with infective complications [1, 3]. Also, there were no definite ischaemic complications reported for GDA/PDA (gastroduodenal artery/pancreaticoduodenal artery) embolisation. In a post pancreatoduodenectomy patient, pseudoaneurysm formation in a short GDA stump can be a difficult problem to treat with end embolisation alone. Hur et al. [35] demonstrated a high rate of rebleeding following the embolisation of the GDA stump and pseudoaneurysm. Even when the CHA also was embolised proximal and distal to the GDA stump, the risk of further bleeding remained significant.

This raises the issue of the safety of embolisation of the CHA and vessels distal to it. Hur et al. [35] found a high incidence of hepatic infarction (3/16, 19 %) with this procedure. Although they were able to manage all their cases conservatively, outcomes following embolisation of the hepatic arteries were not as favourable in the other studies reviewed. In the few studies where specific details of the embolised vessels and the clinical outcomes were available, a total of 26 proper hepatic artery (PHA)/CHA and 10 right/left/middle hepatic artery (R/L/MHA) embolisations were reported [3, 5, 12, 13, 15, 27]. In those patients, there were seven instances of hepatic failure (of which six died), ten with hepatic infarction and three with a liver abscess. Overall, the morbidity rate was 56 % and the hepatic failure rate of 19 %. In another recent study of hepatic artery embolisation, 23 % (6/26) of patients developed liver infarction or an abscess. Sato et al. [36] also reported a high morbidity rate (45 %), hepatic failure rate (47 %), and mortality rate (30 %) following hepatic artery embolisation for bleeding after major pancreatic and hepatic surgery. These authors stress the importance of collateral vessel formation requiring multiple embolisations, which increases the risk of fatal complications.

Pseudoaneurysms arising from the SMA are uncommon. These can be managed operatively or by radiological placement of a stent. Miura et al. [5] reported a single case of coil embolisation of an SMA pseudoaneurysm where they managed to maintain patency of the SMA. Unfortunately, the patient subsequently rebled and died. Lee [15] described a patient in whom a SMA stent resulted in thrombosis and the ensuing intestinal ischaemia caused the patient to be dependent on parenteral nutrition. Two cases of SMA pseudoaneurysmal bleeding treated by stent deployment demonstrated control of the bleeding, although one patient died from sepsis due to chronic stent infection [34]. Intra-arterial infusion of vasopressin as another radiological treatment option for pseudoaneurysms arising from the SMA has been described with a successful outcome [13]. However, treating a structural anatomical abnormality with a temporary vasospasm agent would seem inadequate.

### Rebleeding and the role of definitive surgery

Rebleeding after embolisation can occur early or late. Early failure may be due to technical problems (e.g., failure to cannulate or localise the bleeding) or to complications during the procedure (e.g., arterial perforation or dissection by the catheter) [14]. From the data presented, a significant proportion of patients rebled at a later date despite an initial successful embolisation (Table 2). Given that the initiating event of the pseudoaneurysm (pancreatic enzymes, local inflammation, or anastomotic leaks) is not dealt with by embolisation, it is likely that these factors contribute to the late rebleeding. While some studies specifically reported 12- or 24-h rebleeding rates, there was great variation in reporting, and in many cases it was difficult to discern between a rebleeding episode and primary failure. When rebleeding was reported, only a few studies provided details about the timing of the bleed and the original presentation or associated pathology. Overall, however, it was clear from the data available that the risk of rebleeding is present days and even months after an initial successful embolisation [14, 24, 30].

Although the data are heterogeneous, several interesting observations can be made from the studies reviewed. First, routine imaging within the first week of the initial embolisation often detects filling of a residual pseudoaneurysm. Sethi et al. [27] used CT scan follow-up at 24 h and at 1 week. They observed contrast in the aneurysmal sac in 20 % (3/15) of their cases despite haemodynamic stability and no clinical evidence of ongoing haemorrhage. The presence of such a residual pseudoaneurysm may contribute to the risk of late bleeding despite apparent initial successful haemostasis. Second, the underlying secondary pathology such as a pseudocyst or a GIT anastomotic leak may increase the risk of rebleeding. This may be related to exposure of the vessels to degradative enzymes such as the lipase-rich fluid in a pseudocyst. This was demonstrated in a small study of patients with severe pancreatitis in which there was a rebleeding rate of 40 % (2/5) with a pseudocyst but only 20 % (1/5) in those without residual fluid collections [23]. A similar relationship appears to be true also in patients with pancreatitis. There were two studies in which the underlying pathology was specifically treated at or around the time the bleeding was controlled. In the study by Gambiez et al. [37], definitive surgery was performed on most patients at the time of the initial bleeding presentation; this resulted in no rebleeding after a median follow-up of 60 months. Udd et al. [25] treated all pseudocysts endoscopically if they were still present at 6 months and found no rebleeding at the 1-month follow-up.

Of course, there are delayed complications other than rebleeding that can occur after initial control of the bleeding pseudoaneurysm. They are related to the ongoing pathology as well as foreign body (coils or stents) placement. Carr et al. [38] described 3/16 patients with pancreatitis treated for a pseudoaneurysm who developed late complications. One patient required drainage for an infection of a thrombosed pseudocyst and two others had problems with coil migration into the left and right hepatic arteries, respectively, causing left lobar infarction in one. This highlights the importance of investigating and treating any associated pathology as well as dealing with the bleeding pseudoaneurysm. These patients are often unstable and require prioritisation of treatment, usually by controlling the bleeding first, resuscitation second, and then a planned approach to fixing the precipitating pathology. The timing of endoscopic or surgical management of a pseudocyst, or operative intervention for an anastomotic leak, is often difficult because of sepsis or malnutrition. These patients are best managed in a tertiary institution by a multidisciplinary team in a high-dependency or intensive care environment.

**Fig. 1:**
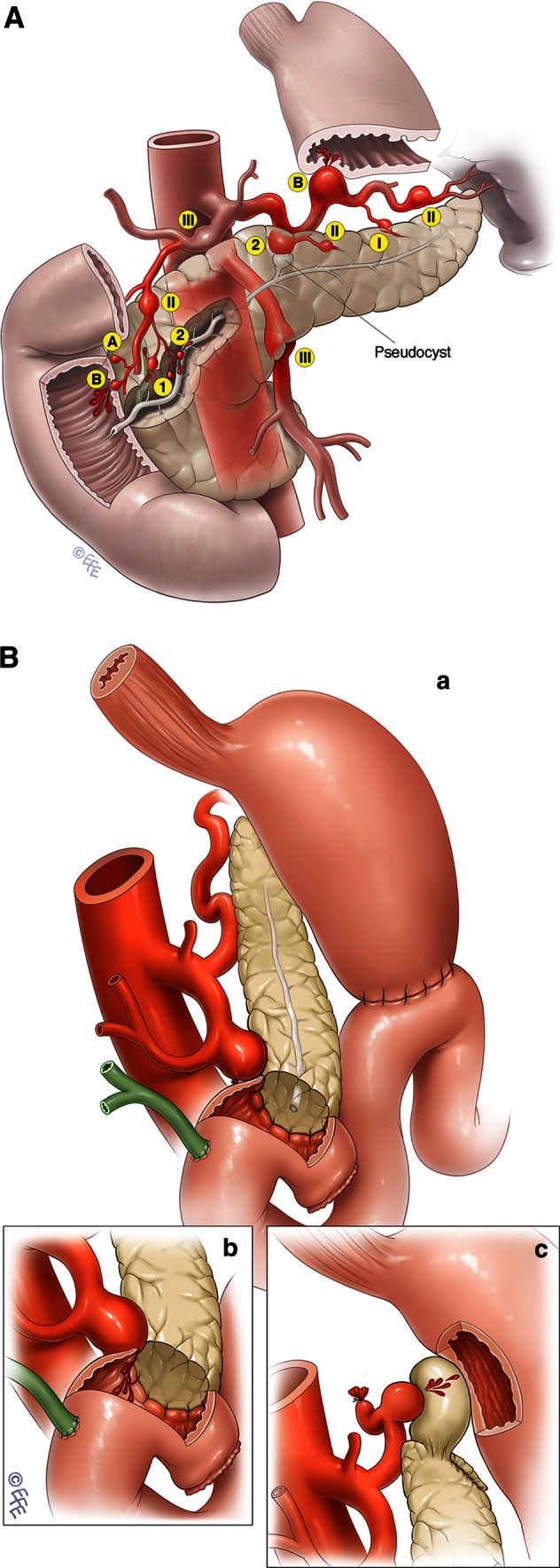
*Top* Examples of the proposed pseudoaneurysm classification based upon the artery type (*I*–*III*), communication with GIT (*A*, *B*), and exposure to pancreatic juice (*1*, *2*). *Bottom* Further specific examples of pseudoaneurysms arising after pancreatic surgery. **A** A pseudoaneurysm originating from a short GDA stump may arise as a result of a leak from the adjacent pancreatic anastomosis; this is a type IIIB2 aneurysm. **B** Such a pseudoaneurysm, if it ruptures into the anastomosis, will cause massive gastrointestinal bleeding. **C** Rupture of a splenic artery pseudoaneurysm into a pseudocyst arising from a pancreatic leak after distal pancreatectomy; this is a type IIA2 pseudoaneurysm

Although embolisation has made a dramatic impact on the management of acute bleeding from peripancreatic pseudocysts, radiological management may only be a bridge treatment for some patients. It would be ideal to be able to distinguish a patient as being in one of three groups at the time of presentation: those that can be successfully treated with embolisation alone without the risk of delayed rebleeding, those in whom embolisation may provide only a bridge to possible further surgery, and those who will require early endoscopic or surgical intervention. A management classification of peripancreatic pseudoaneurysms is proposed, based on the following factors that are identified after appropriate imaging (Table 4; Fig. 1A): (1) the vessel of origin, (2) the presence or absence of communication with the GIT, and (3) the presence or absence of pancreatic juice at the bleeding site. The first factor influences the selection of the initial haemostatic strategy (embolisation, stent, of surgery), while the other two factors may influence the decision for definitive endoscopic or surgical management (Fig. 1B).

The arteries from which the pseudoaneurysm arises can be classified into three groups:

1. type I arises from a minor artery but must be at least 5 mm away from its junction of origin from a type II or III artery
2. type II arises from a major artery that may be sacrificed without physiological consequences (e.g., splenic and gastroduodenal arteries)
3. type III arises from a major artery that cannot be sacrificed without significant consequences for the patient (e.g., the SMA or the hepatic artery proper)

Pseudoaneurysms can be further subclassified into:

- type A where there is no communication with the GIT
- type B where there is a communication with the GIT

In type A, bleeding tends to be low volume, often creating a haematoma within a confined space. In the retroperitoneal space, the expanding haematoma results in rising pressure, which usually compresses the bleeding site. These patients may develop acute renal failure as a result of an abdominal compartment syndrome [39]. In type B, patients frequently have a sentinel bleed that may be followed by a catastrophic haemorrhage. Acute bleeding can be controlled by either embolisation or a stent, but these patients possibly have a higher risk of rebleeding and infection as a result of contamination from the GIT. A more definitive surgical procedure to deal with the pseudoaneurysm may need to be considered after haemodynamic stabilisation with embolisation or an endovascular stent. In patients in whom aneursymal coils and glue or an endovascular stent is exposed to a significant amount of GIT contents, the risk of infection could lead to rebleeding. This risk of rebleeding has to be balanced against the risk of surgical intervention. A long and narrow communication in an elderly patient can be observed, while a short and wide communication in young patient may need a more definitive surgical approach.

We suggest a further subclassification according to exposure to pancreatic juice:

i. type 1 is no exposure to pancreatic juice
ii. type 2 is exposure to pancreatic juice

In a type 2 pseudoaneurysm, enzymes within the pancreatic juice can chemically digest the artery wall. This may lead to further pseudoaneurysm formation or a breaking of the seal between the native arterial wall and the stent or embolisation agent. Patients with a pseudoaneurysm and pseudocyst following pancreatitis and endoscopic transgastric drainage of a pseudocyst may convert type 2 into type B. Some of these patients then can be managed conservatively while others may require more definitive surgical management in the long run. The main cause of a pseudoaneurysm following pancreatic surgery is pancreatic fistula. In some patients with an ISGPF type C pancreatic fistula [41] and a pseudoaneurysm, completion pancreatectomy can salvage the situation [42]. Others may have to be managed by diverting the pancreatic juice away from the pseudoaneurysmal vessel.

In summary, peripancreatic pseudoaneurysms may be classified by a combination of the above factors as summarised in Table 5.

**Table 5:** Peripancreatic pseudoaneurysm classification system

Type of artery		Communication with GIT		Exposure to pancreatic juice	
I	Minor artery >5 mm away from major artery	A	No Communication	1	No exposure
II	Major artery which may be sacrificed	B	Communication	2	Exposure
III	Major artery which cannot be sacrificed				
Examples: Splenic artery pseudoaneurysms (type II) arising from a pancreatic pseudocyst (type 2) with no communication with GIT (type A) are classified as type IIA2 pseudoaneurysm A GDA stump (<5 mm) pseudoaneurysm arising from an area of enteropancreatic leak is classified as a type IIIB2 pseudoaneurysm.					

## Conclusion

Peripancreatic pseudoaneurysm formation is a major complication that can result following pancreatobiliary surgery or pancreatitis. It is frequently associated with significant morbidity and mortality. Patients who form a peripancreatic pseudoaneurysm are at high risk of rebleeding and often require a definitive surgical procedure after initial radiological control by embolisation or placement of a stent. Clinicians need to be aware of the risk of rebleeding at the onset of treatment as this can have a significant impact on the definitive treatment strategy. We propose that the risk of rebleeding depends on infection and chemical digestion. The former is often due to communication with the GIT and the latter is caused by direct contact between the arterial wall and pancreatic juice. The management of this condition is usually a combination of radiology, endoscopy, and surgery and this combination may be assisted by a logical classification system.
